# Quantifying ‘surprise’ in capuchin monkeys (*Sapajus apella*)

**DOI:** 10.1098/rsos.260264

**Published:** 2026-11-04

**Authors:** Gabriella E. Smith, Megan L. Lambert, Amanda M. Seed

**Affiliations:** ^1^ Animal Innovation Research Unit, Messerli Research Institute, Department of Interdisciplinary Life Sciences, University of Veterinary Medicine Vienna, Medical University of Vienna, University of Vienna, Vienna, Austria; ^2^ School of Psychology and Neuroscience, University of St Andrews, St Andrews, UK

**Keywords:** violation of expectations, information seeking, capuchin monkey, surprise

## Abstract

Violation-of-expectation (VoE) events offer a fascinating lens into the intersection of cognition and emotion. Research suggests that, like humans, many animals are susceptible to VoE events in many physical domains, susceptibility typically assessed through behaviours such as visual and physical exploration of a stimulus. In humans, such susceptibility has been further documented through emotional surprise, such as delight following positive surprise and frustration following negative surprise. However, evidence in animals remains relatively limited. To address this gap, this study examined behavioural, cognitive and emotional responses of capuchin monkeys (*Sapajus apella*) to bait-and-switch VoE events of food items. Capuchins were chosen for this study not only for their exploratory and information-seeking behaviours, but also for known emotional responses to VoE events (e.g. frustration following inequity aversion). Contrary to prior research, this study found no evidence for violation susceptibility via behavioural, cognitive or emotional responses. Overall, such findings contribute valuable information to the VoE literature in primates regarding methodology and information salience, as well as underscore the current challenges of investigating emotional surprise in animals. Future research may benefit from establishing stronger expectations (e.g. more salient stimuli), to better investigate and quantify surprise to VoE events in animal species such as capuchin monkeys.

## 1. Introduction

Violation-of-expectation (VoE) events offer a valuable lens into one's perception of the world. Critically, VoE events occur when one's core knowledge about the environment is violated, a phenomenon psychology has historically harnessed to examinequestions into cognition (see [1] for a review). For example, infants will not only look longer at objects that violate expectations (e.g. solidity; individuation; permanence [2–5]; see [6] for a review) but will also explore them in ways consistent with the specific property violated (e.g. drop objects that float instead of fall; bang objects that pass through solid walls [7]). Emotional responses to VoE events have also been examined in humans, with infants tending to respond with elation to positive VoE events (e.g. laughter at an absurd action [8]) or frustration to negative VoE events (e.g. banging a barrier blocking access to once-accessible toys [9]). Termed the elation and frustration effects [10], respectively, such emotional responses to surprise may have evolved to release the tension built by met or unmet expectations [11,12] and/or communicate information about the valence of environmental changes to conspecifics (see emotional contagion [13]).

Surprise research in non-human animals (hereafter, animals) also uses VoE paradigms to examine behavioural, cognitive and emotional responses, though the latter remains relatively understudied. Similarly to humans, animals visually and physically explore objects longer that violate expectations of physical rules such as continuity and support relations (e.g. dogs [14]; rooks [15]) as well as object permanence and individuation (e.g. grey parrots [16]; kea parrots [17]; macaques [18]). Such research suggests that many species maintain expectations about the physical world similarly to how humans do, but how animals respond to VoE events emotionally remains more unclear. Some animal studies report frustration following negative VoE events (e.g. aggression and/or rejection of low-value food when expecting high-value food [16,18]), whereas others do not [19]. However, evidence for elation following positive surprise (e.g. delight upon finding low-value food swapped for high-value food) is even more rare. The only cases of these originate from incentive contrast research in which rodents ran faster on a runway following a reward increase relative to prior trials [10,20] (but see [21]). Critically, evidence for the elation effect in animals has not been documented since (e.g. [19,22,23]).

This study serves as further exploration into animals’ cognitive and emotional responses to VoE events in the model species, the capuchin monkey (*Sapajus apella*). A subfamily of simian primates, capuchins are known for their sophisticated physical cognition skills [24,25], visual [26] and tactile exploratory tendencies [27,28], as well as susceptibility to sleight-of-hand tricks [29] and violations in object permanence and individuation [30,31]. With regard to emotional responses following VoE events, capuchins have been observed to exhibit frustration when conspecifics receive rewards of higher value for performing the same task but do not exhibit elation when the opposite occurs (inequity aversion [32]; but see [33]). This study thus aims to examine capuchins’ behavioural, cognitive and emotional responses to bait-and-switch VoE events (i.e. surreptitious swap of an expected target for another) of object permanence and individuation varying in positive and negative directions. Based on findings from prior VoE research (e.g. [14,17]), we similarly predict that if capuchin subjects are susceptible to the violation, they will physically investigate (i.e. explore) violating stimuli more than non-violating stimuli during and after the experiment and exhibit behaviours akin to the frustration effect (e.g. rejection of food [16,18]) more following negative violations. With regard to the elation effect, how subjects may respond to positive violations remains difficult to predict owing to lack of current evidence; however, any potential emotional behaviours (e.g. vocalizations [34,35]) occurring during violation contexts will be recorded.

## 2. Methods

This study's stimuli and methods were adapted from Smith *et al.* [17].

### 2.1. Subjects

From December 2023 to January 2024, 19 captive tufted capuchin subjects (*S. apella*) (7F, 12M; supplementary material, table S1) were tested in visually isolated testing cubicles (0.5 × 0.5 × 0.5 m^3^ each, figure 1), to which they were already habituated. Study subjects live in two separate groups (East and West) at the Living Links to Human Evolution, Edinburgh Zoo, Edinburgh, Scotland, United Kingdom (figure 2). Both groups have both outdoor (approx. 900 m^2^) and indoor (7 × 4.5 × 6 m^3^) sections of their enclosures in addition to the respective indoor research rooms with testing cubicles.

**Figure 1.**
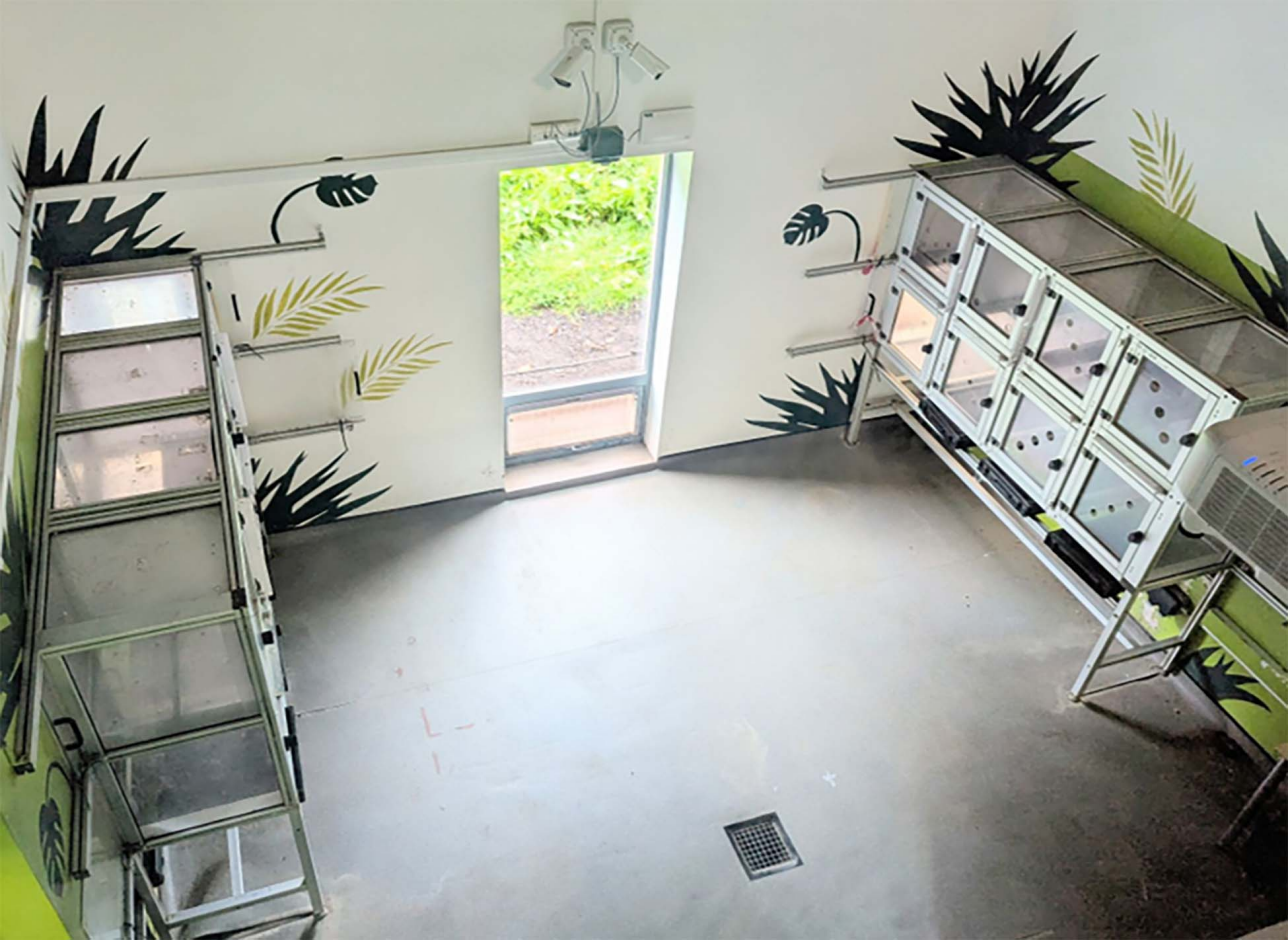
One of two indoor testing cubicle rooms. Photo courtesy of Kate Grounds.

**Figure 2.**
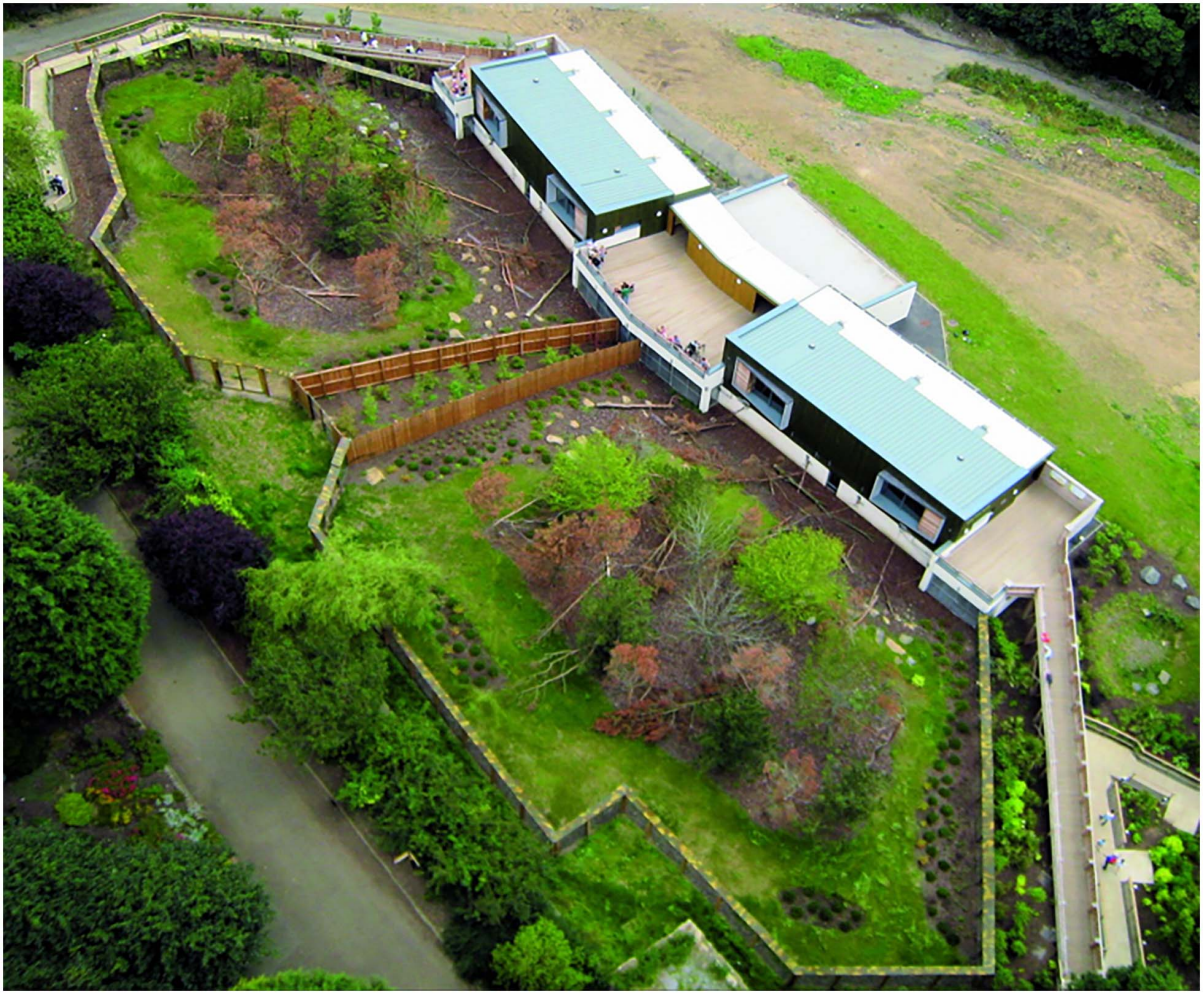
East and West outdoor and indoor enclosures. (Photo courtesy of Living Links).

It is important to note that 15 of the 19 Living Links capuchins studied here experienced a bait-and-switch VoE experiment before. In Kersken *et al.* [30], subjects were found to both physically and visually explore an apparatus more after food was dropped inside and a different food of equal value was revealed. We do not believe this prior experience influences the present study for several reasons: the apparatus in Kersken *et al.* [30] was substantially larger than those in the present study, so this difference in design, plus the 7-year gap in data collection between experiments, makes generalization unlikely; in the prior study, the capuchins were always provided the original, dropped food reward after the violation reveal from behind the apparatus, suggesting a broken mechanism rather than a violating quality of the apparatus. Overall, Kersken *et al.* [30] reinforce prior work documenting object permanence and individuation skills of capuchins (e.g. [31]) and provide a strong foundation to examine the emotional responses to violations of varying directions.

### 2.2. Stimuli

The experimental apparatus consisted of two pairs of wooden towers (8 × 6 × 6 cm^3^), each pair consisting of one white tower and one black tower. In one pair, the towers were hollow, while in the other, clear plastic shelves could catch objects dropped from above. All four towers also had a single clear side each from a previous experimental design but did not influence the present study.

During experimental trials, a single tower was placed on top of a wooden block (10 × 10 × 5 cm^3^) fixed to a wooden plate (26 × 26 × 2 cm^3^). Inside the block, a circular cavity (5 × 3 cm^2^) was filled with wood shavings onto which food was baited at the beginning of each trial (see figure 3).

**Figure 3.**
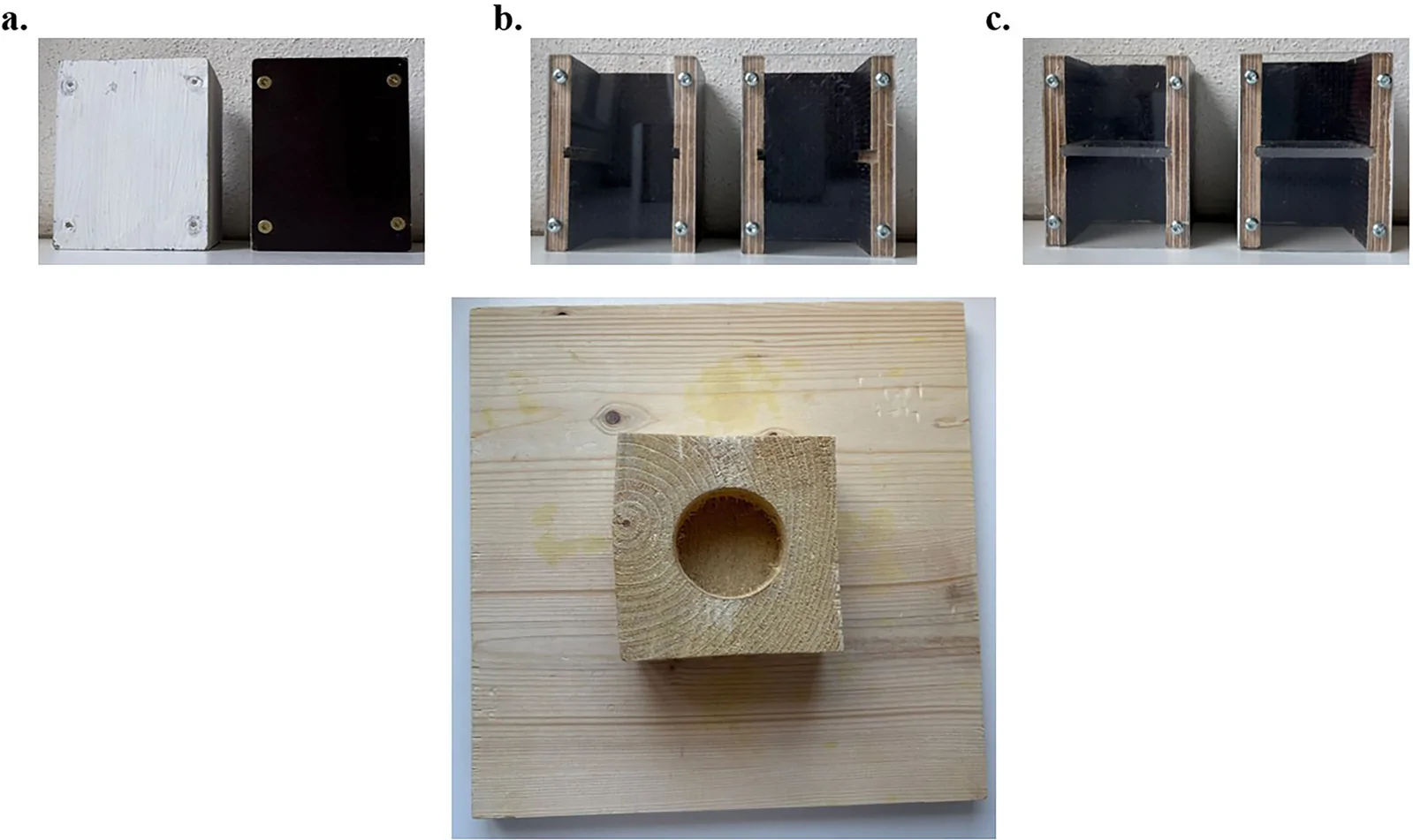
(a) Tower pairs with one white and one black tower each. (b) One pair, used in exploration trials, were hollow with no shelves; (c) the other pair, used in demonstration and test trials, had shelves to catch dropped food; and (d) in experimental trials, a single tower sat on wood shavings in the depression of the wooden plate.

### 2.3. Procedure

#### 2.3.1. Food preferences

Subjects were tested individually on a voluntary basis. Prior to the experiment, subjects experienced three sessions of 12 food preference tests by choosing one of two food rewards they are known to eat willingly (raisin, grape and maize; see supplementary material, table S3). Chi-square tests determined individual high, medium and low values of food preferences prior to experimental trials (see below).

#### 2.3.2. Exploration and experimental trials

Subjects were given two, unrewarded exploration periods of the shelf-less pair of towers (left–right placement randomized between individuals), one before and one after experimental trials (figure 4). Subjects were allowed to freely interact with the towers for a maximum of 30 s, but exploration periods were stopped as soon as a subject did not interact with the towers for at least 20 s. After the first exploration period, two demonstration and four experimental trials followed with the shelved set of towers. At the start of each demonstration or experimental trial, a cardboard barrier was placed in front of a subject to hide stimulus preparation (e.g. pre-bait wood shavings with food) and removed to begin the trial. In demonstration trials, a single piece of individuals’ medium-value food was dropped into the top of the tower sitting on the wood shavings (tower/colour trial order randomized between subjects). The tower was then removed, and an identical, pre-baited food was revealed on the wood shavings below (figure 5). These trials served to demonstrate to subjects that the towers were ostensibly hollow and that objects could pass straight through. Subjects had a maximum of 30 s to freely access the wood shavings after tower removal, and a trial was stopped as soon as a subject did not interact with the towers for at least 20 s. Trial times were extrapolated from Smith *et al.* [17] to the testing conditions at Living Links, namely, institutional restrictions on how long individual capuchins can be tested at a time (i.e. 10 min).

**Figure 4.**
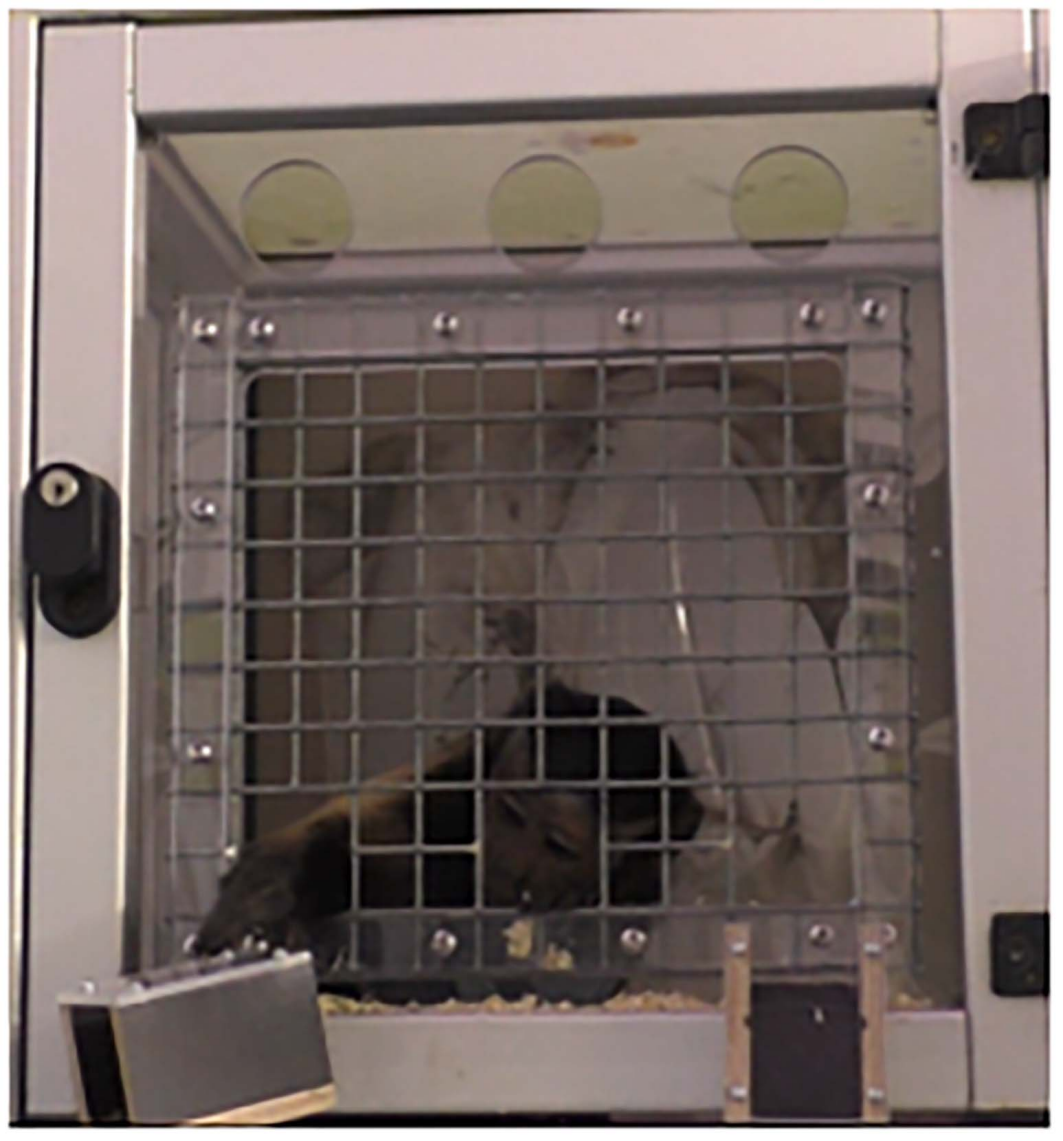
Pixie investigating the shelf-less towers in Exploration 1 (black left, white right).

**Figure 5.**
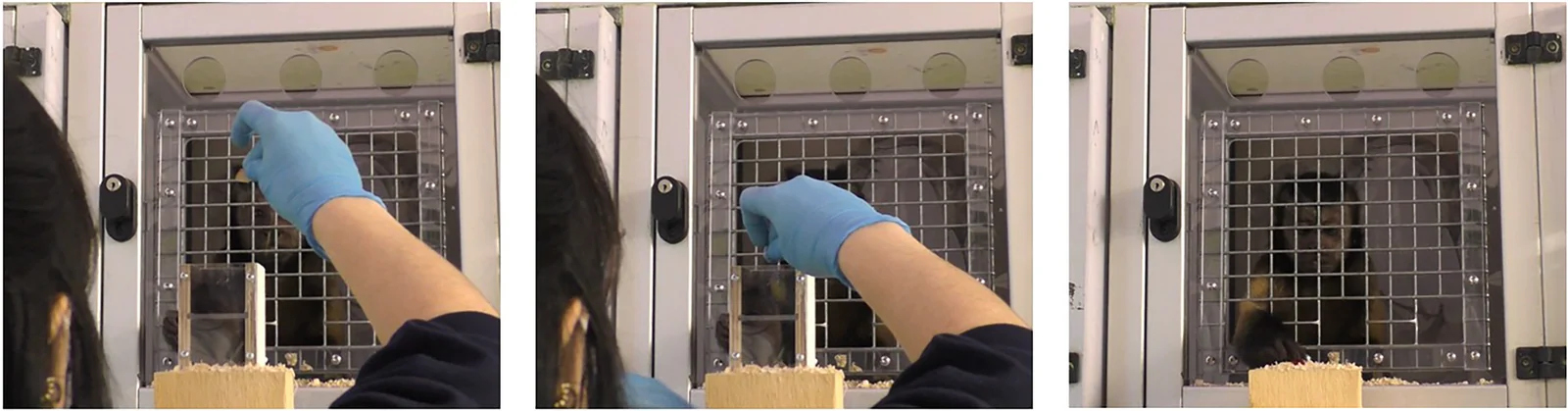
Inti observing an initial demonstration trial and retrieving pre-baited, identical food.

In experimental trials, the same procedure was employed with high- and low-value foods of the same size. The order of these trials consisted of non-violation/violation/violation/non-violation, wherein a non-violation trial involved no change in food identity between the dropped and revealed food, while a violation trial could involve a positive change (low-value-to-high-value) or negative change (high-value-to-low-value) between the dropped and revealed food. Critically, white or black tower colour was always associated with violation or non-violation trials, respectively (colour assignment randomized between subjects). For example, a subject could experience the following order of experimental trials: (1) a maize dropped into the white tower with a maize revealed on the wood shavings (non-violation, low-value-to-low-value); (2) a raisin dropped into the black tower with a maize revealed on wood shavings (violation, high-value-to-low-value); (3) a maize dropped into the black tower with a raisin revealed on wood shavings (violation, low-value-to-high-value); and (4) a raisin dropped into the white tower with a raisin revealed on wood shavings (non-violation, high-value-to-high-value). Subjects had 30 s to retrieve the food and access the wood shavings at-will, and a trial was stopped as soon as a subject did not interact with the set-up for at least 20 s.

Critically, in both demonstration and experimental trials, subjects’ visual attention to the food was determined by gaze direction prior to its release. Furthermore, note that food was dropped close to the top of the tower and done so gently as to avoid excessive auditory feedback from the shelf. However, as subjects never visually observed the shelves in demonstration and experimental trials—and ostensibly considered the towers hollow from the initial exploration—we did not expect any auditory feedback to influence response behaviour.

### 2.4. Data collection and analysis

#### 2.4.1. Video recording and coding

Trials were recorded with a JVC Panasonic camcorder, and behavioural coding was done using BORIS [36]. In exploration trials, which tower was touched first was coded as well as the duration of tower interaction; in test trials, whether subjects ate the food was coded as well as the latency to eat, in addition to duration spent digging in the wood shavings, vocalization counts and interactions with the wood shavings plate (see supplementary materials, table S2 for ethogram).

#### 2.4.2. Statistical analysis

All analyses were performed in the statistical computer software R v. 4.4.2 [37]. For test trials, we were interested in the effects of condition (violation versus non-violation) and its interaction with final food value (high versus low) on subjects’ behavioural responses (see [17]): (i) duration of digging and (ii) latency to eat the reward. As all subjects dug on every trial, we did not examine digging probability (see [17]). Digging duration was modelled with a generalized linear mixed model (GLMM) with a gamma error distribution and log link, including the log-transformed trial time as an offset term to account for differences in trial durations. Eating latency was log transformed and modelled with a linear mixed-effects model with a Gaussian error structure. To avoid cryptic multiple testing, we began by comparing full models (including all predictors) to null models excluding both condition and final food value [38].

For exploration trials, we tested the effect of exploration trial order (pre- or post-test) on subjects’ cognitive responses, namely, whether they would target their exploration towards the towers to seek information about the violation itself (see [14]): (i) probability of first touching the violation tower; (ii) proportion of time spent interacting with the violation tower; and (iii) total duration of interaction with the towers overall. The probability of first touching the violation tower was modelled with a mixed-effect regression with a binomial error structure, the proportion of time interacting with the violation tower was modelled with mixed-effect beta regression, and the duration of time interacting with towers was modelled with a GLMM with a Tweedie error structure and log link, with log trial time included as an offset to account for variable trial durations. Age was included as a control predictor for the latter two models, but sex was not owing to prior literature suggesting little effect of sex on capuchin physical exploration [39,40]. The significance of individual fixed effects was tested by removing them one at a time using the drop1 function and comparing each reduced model to the full model via likelihood ratio tests.

Subject was included as a random intercept effect in all models to account for repeated measures and avoid pseudoreplication. The models with Gaussian or binomial error distributions were fitted using the ‘lmer’ and ‘glmer’ functions of the ‘lme4’ package [41]. Initial models included all possible identifiable random slopes and their correlations with intercepts [42,43]. All other models were fitted using the function glmmTMB (package: glmmTMB [44]). In cases of convergence or singularity issues, we first removed correlations among random intercepts and slopes and subsequently removed random slopes with the lowest estimated variance. Model stability was estimated by excluding levels of the random effects one at a time [45]. We assessed collinearity using the package ‘car’ and found the variance inflation factor to be at or near one for all models, indicating no issues with collinearity. We also included count descriptives of emotional behaviours between violation versus non-violation trials of whether food was eaten (see [16,18]), whether the sand-plate was pushed/slammed (see [32]) and vocalizations (see [34,35]).

Inter-observer reliability for food eating (Y/N), digging duration and tower exploration duration was carried out on 26% of all subjects’ videos between two independent observers.

## 3. Results

### 3.1. Food preferences

Of the 19 capuchins tested, 18 significantly preferred raisin as the high-value reward, grape as the medium-value reward and maize as the low-value reward, while 1 significantly preferred grape as the high-value reward, raisin as the medium-value reward and maize as the low-value reward (see supplementary material, table S3).

### 3.2. Behavioural

During test trials, all subjects dug on every trial and spent an average of 4.96 (±2.41) s digging. Our full-null model comparison was not significant, indicating no evidence for an effect of condition or its interaction with final food value on digging duration (*χ*^2^(3): 0.78; *p* = 0.85) (figure 6; supplementary material, table S4). Of the 19 subjects, 13 consumed the food reward on every trial. Two subjects did not consume the reward in the non-violation condition when the food value was low, and four subjects did not consume the reward in both the violation and non-violation conditions when the final reward value was low. Over all trials, subjects took an average of 4.65 (± 5.79) s to access the reward. For latency to eat the reward, the full model fit the data significantly better than the null model (*χ*^2^(3): 17.95; *p* < 0.001) (figure 7). Subsequent model estimates showed that subjects had significantly higher latencies when the final food value was low, whereas neither condition nor its interaction with final food value had a significant effect (supplementary material, tables S5 and S6).

**Figure 6.**
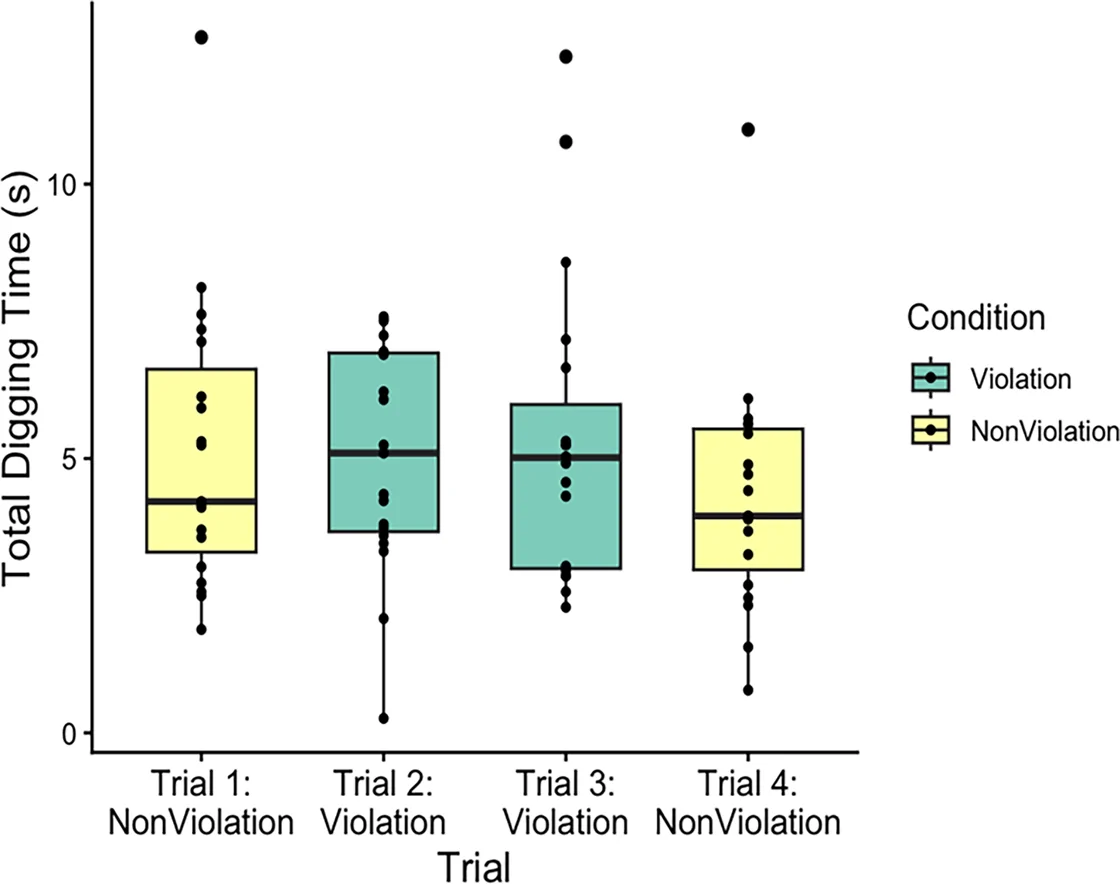
Digging durations across the four experimental trials. Box plots represent the median (horizontal line inside the boxes) and interquartile range (IQR: outer box borders), with whiskers extending to 1.5× the IQR. Individual points represent each subject.

**Figure 7.**
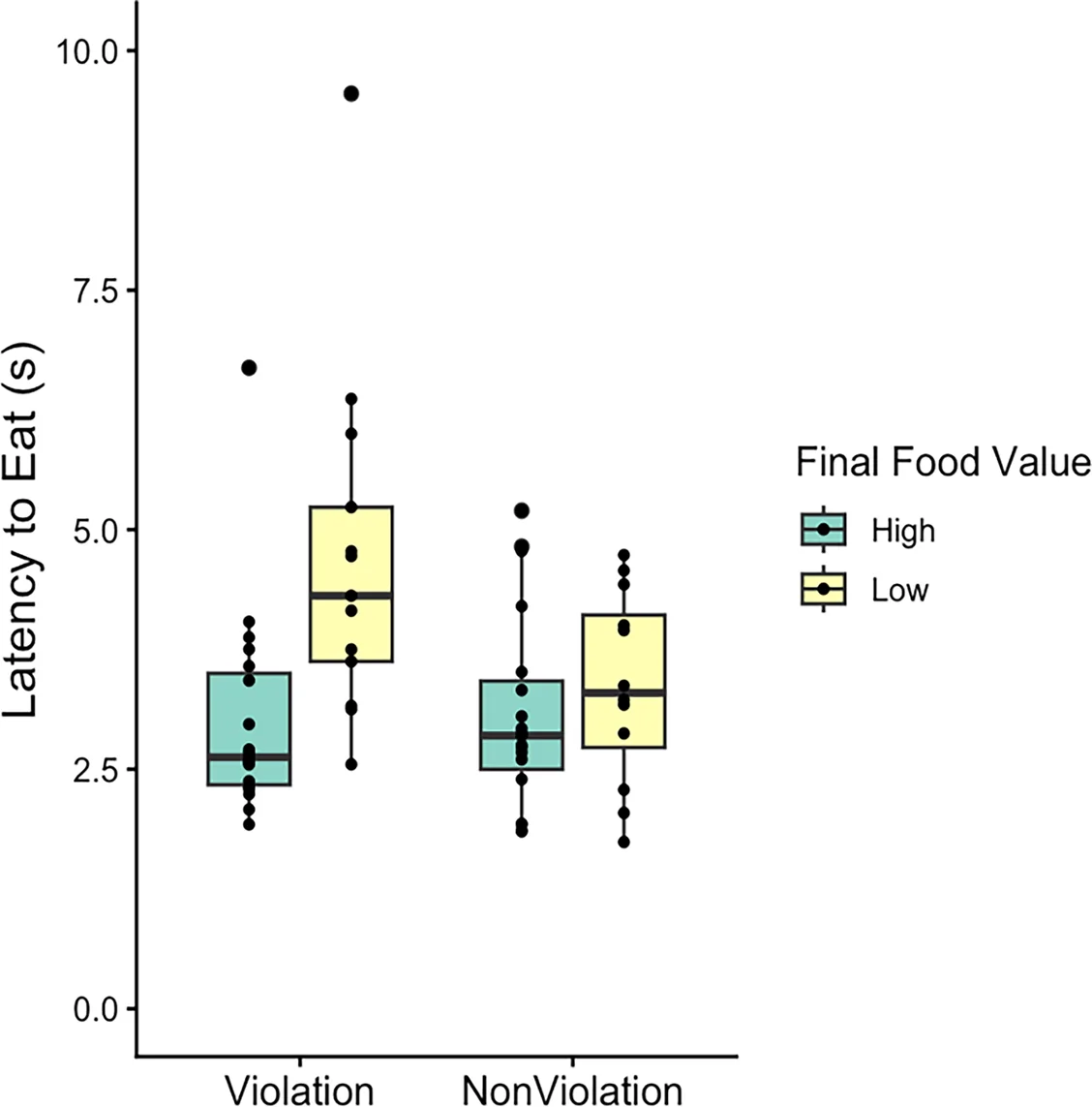
Latencies to eat food in violation and non-violation trials. Box plots represent the median (horizontal line inside the boxes) and interquartile range (IQR: outer box borders), with whiskers extending to 1.5× the IQR. Individual points represent each subject. Note that three outliers have been excluded for visualization purposes only (two values of 32 and 24 s in violation, final food value: low; and one value of 32 s in non-violation, final food value: low).

### 3.3. Cognitive

During exploration trials, 12 subjects interacted with at least one tower during the first exploration trial, and 16 subjects interacted with at least one tower during the second exploration trial. Subjects spent an average of 8 (±7.6) s exploring the towers in both exploration trials overall (figure 8). There was no significant effect of exploration trial number (1 or 2) on subjects’ probability to first touch the ‘violation’ tower, proportion of exploration time directed to the violation tower, nor duration of overall tower exploration (figure 9; see supplementary material, tables S7–S9 for model outputs). However, there was a significant effect of age on overall exploration, with younger individuals exploring towers more than older individuals regardless of exploration trial (supplementary material, table S9).

**Figure 8.**
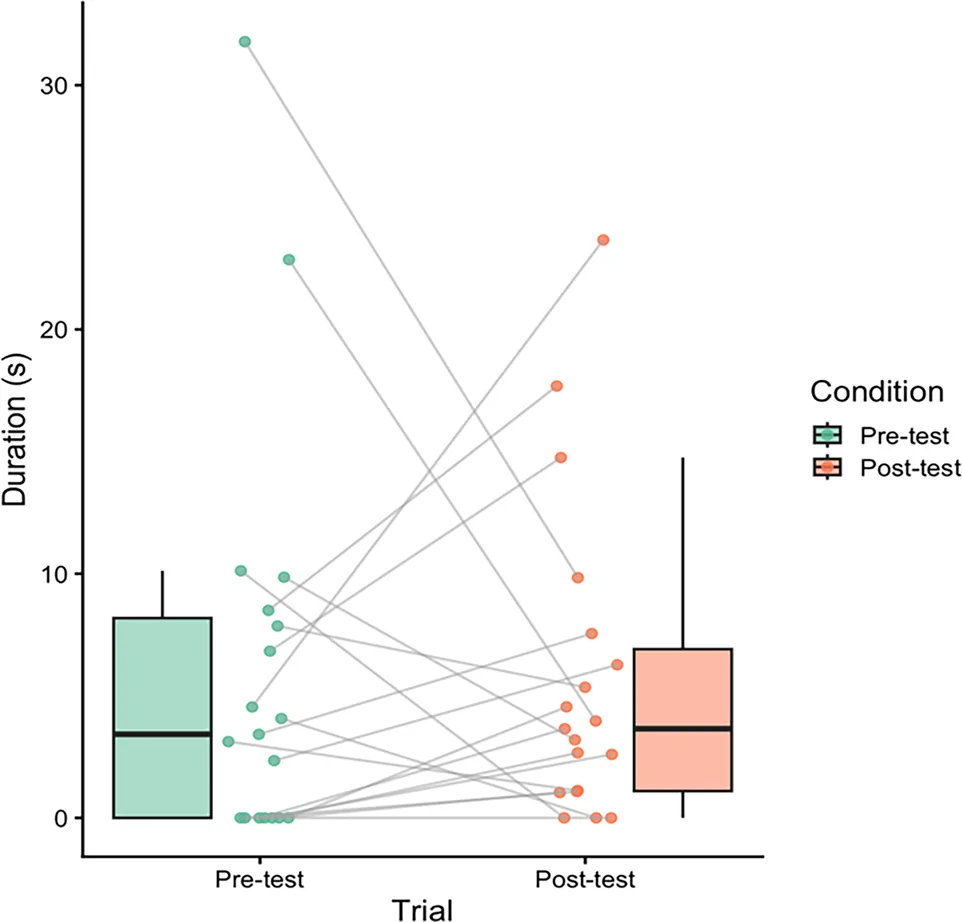
Durations of overall exploration towards either tower across the two exploration periods (pre-test and post-test). Box plots show the distribution of exploration durations, with horizontal lines representing the median and boxes representing the IQR. Whiskers extend to 1.5× the IQR, and points beyond this represent outliers. Dots show individual data points, with grey lines connecting repeated measurements from the same individual across exploration periods.

**Figure 9.**
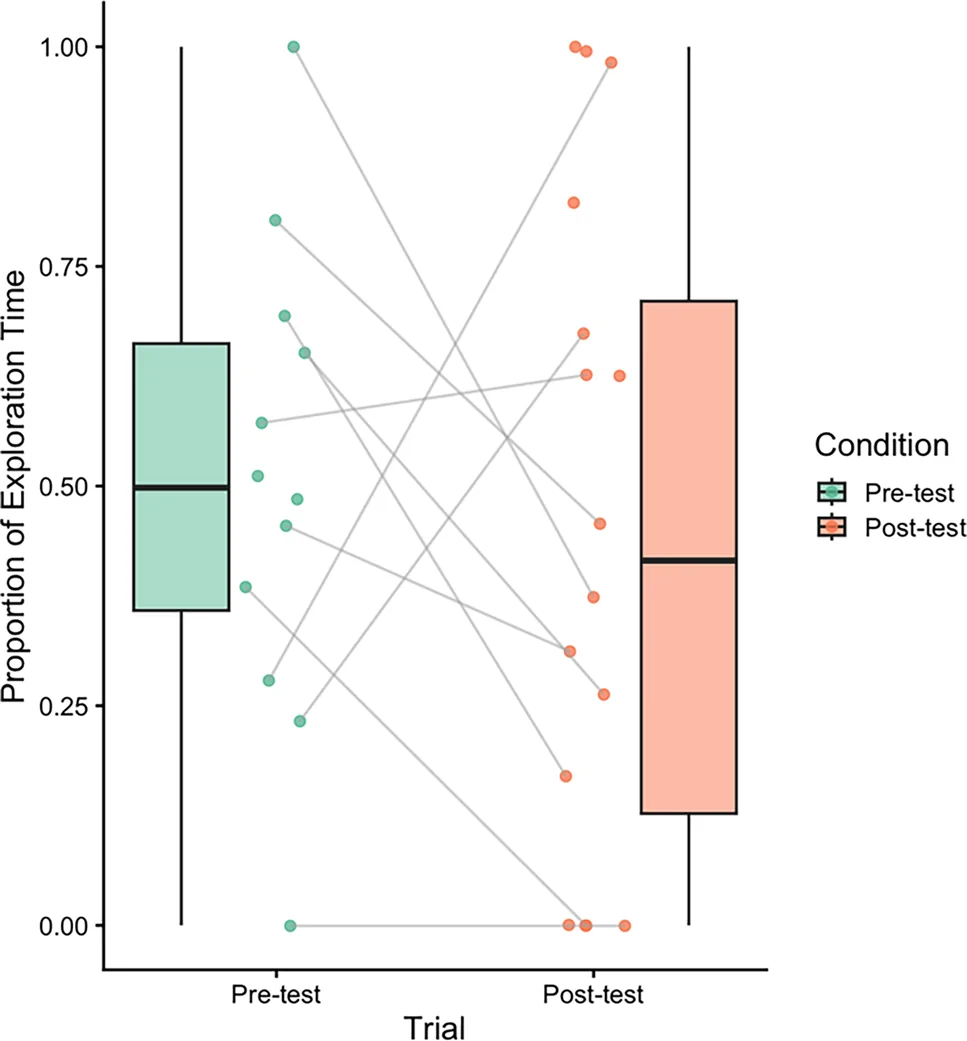
Exploration durations of violation towers as proportions of total exploration times in Explorations 1 (pre-test) and 2 (post-test). Box plots show the distribution of exploration proportions, with horizontal lines representing the median and boxes representing the IQR. Whiskers extend to 1.5× the IQR, and points beyond this represent outliers. Dots show individual data points, with grey lines connecting repeated measurements from the same individual across exploration periods.

### 3.4. Emotional

There were not enough individuals producing elation- or frustration-related behaviours (i.e. vocalizations; sandplate roughhousing; table 1), nor counts of elation- or frustration-related behaviours (supplementary material, table S10) to comment on the effect of the violation on emotional response.

**Table 1.** Counts of subjects expressing at least one frustration/elation behaviour.

behaviour	violation	non-violation
food eating	19	19
vocalizations	11	8
sandplate roughhousing	6	6

### 3.5. Inter-observer reliability

Inter-observer reliability (*n* = 5) for eating (ICC = 0.963), digging duration (ICC = 0.83) and tower exploration duration (ICC = 0.99) were all excellent.

## 4. Discussion

This study examined behavioural, cognitive and emotional responses of capuchin monkeys to VoE events and found no evidence for violation susceptibility via digging duration, food consumption, latency to eat food, nor tower exploration. Similarly, no elation nor frustration effects were observed via emotive behaviours (e.g. aggression and vocalizations).

The results regarding violation susceptibility are particularly surprising. While one could argue that such findings might indicate that our subjects did not link the contingency between food dropped above and discovered below, research into gravity bias in monkeys suggests this is likely not the case. Specifically, gravity bias—the expectation to find a dropped object directly below its drop point despite potential barriers [46]—has been found to actually prohibit monkeys from rationalizing the location of dropped food if it differs from the original drop point [47,48]. Owing to this, the monkeys studied here were likely to expect dropped food directly below its release point.

Such results are also surprising owing to prior literature. Specifically, a 2020 study, including many of the same subjects (15 of the 19 subjects in the present study), found that capuchins do, indeed, respond to violations of property/kind expectations by searching more frequently in violation versus non-violation trials. While we cannot entirely exclude the role of prior experience, we think the 7-year gap in data collection between experiments makes the influence of prior experience unlikely (especially given the very different appearance of the apparatus used in this study), and differences in results may instead stem from methodological or sample-size factors. Similar to the present study, in [30], subjects observed a single food item dropped into a box with a hidden shelf, the bottom containing a same or different reward to explore. However, note that the previous study used a large polycarbonate box (35 × 25 × 40 cm^3^) and this one used much smaller wooden towers (8 × 6 × 6 cm^3^). There was no reason for the monkeys to think that this apparatus would behave like the larger box used in the prior work, given that they have seen a large number of other containers and boxes behave in a way consistent with gravity and object permanence in the interim (e.g. [49–51]). In the prior study [30], subjects had only physical or visual access in their exploration periods, depending on trial condition. In contrast, subjects in the present study had access to *both* visual and physical information during exploration, this combination potentially reducing the extent of discriminate physical exploration following access. In addition, the food rewards of the prior study were presented in an empty and darkened compartment of a box, while the food rewards in the current study were presented in substrate, a set-up potentially prompting more exploration overall regardless of trial type, perhaps owing to past foraging experience. Furthermore, differences in results between these studies may also be explained by sample size. As the previous study included 10 more subjects, it is possible the relatively smaller sample size here did not have the power necessary to detect an effect. Thus, future work could increase the sample size, as well as reference the methods of Kersken *et al.* [30] to include separate visual and physical exploration conditions to more precisely isolate VoE-induced information seeking.

It is important to consider the role of overall novelty or salience when interpreting the results of a VoE. On the one hand, it is possible that if test stimuli are very novel and remain highly salient throughout the experiment, this could distract subjects from the violation at hand (see [52] for a discussion of the use of habituation in VoE experiments). On the other hand, if participants become accustomed to experiencing numerous novel stimuli and experimental apparatuses, the converse effect could arise. Put simply, if subjects consistently experience novel experiments with potentially surprising elements throughout their lives, a VoE might not be salient enough to prompt a significant response. In the case of this experiment, despite our lack of a habituation phase beyond familiarization, it does not seem as if our procedure was overly novel, as exploration was not at ceiling. On the other hand, these capuchins have a lot of experience in cognitive experiments, and even some limited experience with a violation of this kind, and so perhaps the violation was not salient enough to trigger a surprise effect. However, we did observe reasonable levels of exploration, and so it does not seem that the participants were uninterested. Nevertheless, future research may employ more salient elements inherent to the experiment, such as food value, to ensure that the materials are sufficiently engaging (discussed below). In general, the difficulty of calibrating materials correctly considering the experience of study participants is one that deserves further attention, especially when comparing species’ motivation to explore, which is likely to be sensitive to both specific violations, overall novelty and other factors.

With regard to other measures of violation susceptibility recorded here, such as food consumption and latency to eat food, the lack of significant results also contradicts prior research. As mentioned earlier, studies into VoE susceptibility in other species have observed higher latencies to eat, or even rejection of, swapped food following a violation (e.g. [16,18]). A possible reason for results observed here could lie in the food items used as well as the substrate. With regard to the food used, the highest-value reward for most capuchins in the present study was raisins, a reward that all subjects receive daily and frequently. Thus, while raisins are a high-value reward, it is possible they were not rewarding *enough* to prompt food rejection or longer eating latencies of the low-value food when a swap occurred. Furthermore, with regard to the substrate, as it is not uncommon for subjects to find food hidden in substrate in their daily lives (e.g. in zookeeper-provided enrichment), it is possible this contributed to the lack of discriminate food rejection between trial types. Furthermore, the majority's least-preferred reward, maize, resembled the wood shavings more closely than the raisin, so it is possible the higher latencies to retrieve it could have resided in subjects’ inability to immediately detect it in the substrate. Thus, future research could use more rare or more desirable foods—such as dates, which were used by Kersken *et al.* [30]—and exclude substrate on which rewards are presented, to examine VoE susceptibility via measures of food consumption and latency.

In terms of other responses following the VoE, we similarly found no evidence for elation or frustration effects via behaviours such as vocalizations and plate roughhousing. These findings contrast with literature demonstrating frustration responses in capuchin monkeys. For example, Brosnan & de Waal [32] found that brown capuchin monkeys exhibit frustration (e.g. food rejection and refusal to participate) when given rewards of lesser value than conspecifics for the same task, yet display no elation when given better food than that of conspecifics. While violations of social expectations, such as those involved in inequity aversion, may be more likely to prompt emotional responses like frustration in social species than physical violations, it is possible the *interaction* between social context and resource security plays a more prominent role (see also [33]). Put simply, animals may be more likely to exhibit frustration following a negative violation in order to communicate environmental changes (e.g. emotional contagion [13]), but *less* likely to exhibit elation following a positive violation to safeguard valuable findings (see [17]). For example, while macaques have been found to respond aggressively following a negative violation [18], they have also been found to exhibit aversive reactions following positive VoE events [22], which authors attribute to wariness of the better-than-expected reward. While the findings of the latter study are more consistent with expectations of behaviour upon discovering worse-than-expected rewards, it aligns with the suggestion that animals may avoid bringing attention to valuable resources. Thus, future research may benefit from comparing social and non-social paradigms to examine the intersection of social context and resource security on prompting emotional responses following VoE events.

Concerning exploration periods before and after the experiment, there was no significant effect of exploration trial on overall tower exploration, nor on violation quality on specific tower exploration. With regard to the former, however, there was a significant effect of age on overall tower exploration duration, with younger individuals exploring longer than older individuals regardless of exploration trial. This finding coincides with ontogeny literature documenting that younger capuchins overall physically explore more than older conspecifics [53]. Regarding the latter, a possible reason for this could lie in the fact that each subject experienced only one experimental session consisting of four experimental trials (two violation and two non-violation), thus potentially causing weak association between tower colour and violation quality. Furthermore, as the subjects were not found to be susceptible to the violation, it is possible no violation quality was encoded with the towers at all, thus causing no directed exploration in the second exploration period. Despite these possible limitations, however, the inclusion of both stimuli in the exploration period in this experiment offers some advantages as a general approach to the study of information seeking following a VoE event. For example, in the developmental literature, infants have been found to approach and explore a violating object in a choice task with a non-violating object, highlighting specific violation susceptibility rather than overall arousal [54,55]. By providing more than one stimulus to explore, such an exploration period permits the expression of targeted information seeking—a sophisticated cognitive phenomenon requiring the maintenance of specific information in-mind—thus accommodating for the alternative explanation of generalized arousal towards a stimulus caused by the observed violation. Overall, the inclusion of such an exploration period offers a valuable methodological contribution to the growing trend examining the role of exploration in VoE research in animals (see [14,17]).

Overall, this study serves as a first of its kind examining capuchin monkeys’ susceptibility to VoE events via a multifaceted approach. Future research could benefit from testing more subjects, using more sharply contrasting conditions and using the role of the social context to discern VoE perception and emotional response. In this way, we can better understand the intersection of emotion and cognition of non-human animals in VoE events.

## Supplementary Material



## Data Availability

Data is accessible in supplementary material files [56].
