# Supplementary material for: Quantifying ‘surprise’ in capuchin monkeys (Sapajus apella)
Source: R Soc Open Sci. 2026 Nov 4;13(11):260264. doi: 10.1098/rsos.260264 (PMC13730647; doi:10.1098/rsos.260264)
Supplement: Supplementary file 1 [file rsos.260264.zip › rsos.260264/rsos260264_si_001.docx]

**Supplementary Materials**

**Table S1** Individual demographic information, random assignment of violation tower color, and prior VoE experience.

| **Name** | **Side** | **Sex** | **Age (years)** | **Violation tower color** | **Participated in Kersken et al. (2020)?** |
| --- | --- | --- | --- | --- | --- |
| Alba | West | F | 13 | black | Yes |
| Anita | East | F | 27 | white | Yes |
| Carlos | East | M | 18 | black | Yes |
| Cayenne | East | M | 6 | white | No |
| Chico | East | M | 15 | black | No |
| Flojo | East | M | 13 | black | Yes |
| Fudge | West | M | 11 | black | Yes |
| Inti | West | M | 15 | white | Yes |
| Junon | East | F | 24 | black | Yes |
| Kato | East | M | 19 | white | No |
| Lana | West | F | 29 | black | Yes |
| Margarita | East | F | 3 | white | No |
| Matoury | East | F | 6 | black | No |
| Mekoe | West | M | 16 | black | Yes |
| Pixie | West | F | 11 | white | Yes |
| Ruben | East | M | 14 | white | Yes |
| Rufo | West | M | 15 | white | Yes |
| Torres | West | M | 13 | black | Yes |
| Ximo | West | M | 14 | black | Yes |

**Table S2.** Ethogram of coded behaviors in BORIS.

| **Behavior code** | **Behavior type** | **Description** |
| --- | --- | --- |
| Time spent touching white tower | State event | Total time spent touching towers, as well as targeted exploration of assigned violation tower during exploration periods. Serves to compare potential surprise before and after violation (Bräuer, & Belger, 2018; Spelke et al., 1992; Völter et al., 2023; Wilson et al., 2023). |
| Time spent touching black tower | State event |  |
| Digging in sand | State event | Total time spent digging in sand after food reveal. Calculated as measure of surprise (i.e., re-checking behavior) between violation and non-violation trials (Schnell et al., 2021; Tinklepaugh, 1928). |
| Vocalization | Point event | Vocalization counts. Recorded to examine the effect of violation trials/corresponding violation directions on potential elation or frustration effects (Tinklepaugh, 1928). |
| Slamming sand plate | Point event | Counts of slamming/dragging sandplate titled “roughhousing”. Akin to frustration behaviors (e.g., Block & Martin, 1955; Pepperberg et al., 1997; Tinklepaugh, 1928). |
| Dragging sand plate | Point event |  |
| Eaten food? | Point event | Latency and choice to eat food to determine if/when food was rejected as a measure of frustration effect (Schnell et al., 2021; Tinklepaugh, 1928). |

**Table S3** Individual food choice chi square tests to determine preferences.

| *Name* | **Choices** | | | **High vs. medium χ2** | | | **Medium vs. low χ2** | | | **High vs. low χ2** | | | **Preferences*** | | |
| --- | --- | --- | --- | --- | --- | --- | --- | --- | --- | --- | --- | --- | --- | --- | --- |
|  | *High* | *Medium* | *Low* | *χ2* | *df* | *p* | *χ2* | *df* | *p* | *χ2* | *df* | *p* | *High* | *Medium* | *Low* |
| Alba | 19 | 10 | 7 | 2.79 | 1 | 0.09 | 0.53 | 1 | 0.47 | 5.54 | 1 | 0.019 | Raisin | Grape | Maize |
| Anita | 17 | 13 | 6 | 0.53 | 1 | 0.47 | 2.58 | 1 | 0.11 | 5.26 | 1 | 0.022 | Raisin | Grape | Maize |
| Carlos | 22 | 9 | 5 | 5.45 | 1 | 0.02 | 1.14 | 1 | 0.29 | 10.70 | 1 | 0.001 | Raisin | Grape | Maize |
| Cayenne** | 22 | 17 | 9 | 0.64 | 1 | 0.42 | 2.46 | 1 | 0.12 | 5.45 | 1 | 0.020 | Raisin | Grape | Maize |
| Chico | 24 | 9 | 3 | 6.82 | 1 | 0.009 | 3.00 | 1 | 0.08 | 16.33 | 1 | < 0.001 | Raisin | Grape | Maize |
| Flojo | 23 | 12 | 1 | 3.46 | 1 | 0.06 | 9.31 | 1 | 0.002 | 20.17 | 1 | < 0.001 | Raisin | Grape | Maize |
| Fudge | 22 | 14 | 0 | 1.78 | 1 | 0.18 | 14.00 | 1 | < 0.001 | 22.00 | 1 | < 0.001 | Raisin | Grape | Maize |
| Inti | 18 | 16 | 2 | 0.12 | 1 | 0.73 | 10.89 | 1 | 0.001 | 12.80 | 1 | < 0.001 | Raisin | Grape | Maize |
| Junon | 24 | 11 | 1 | 4.83 | 1 | 0.03 | 8.33 | 1 | 0.004 | 21.16 | 1 | < 0.001 | Raisin | Grape | Maize |
| Kato | 21 | 12 | 3 | 2.46 | 1 | 0.12 | 5.40 | 1 | 0.020 | 13.50 | 1 | < 0.001 | Raisin | Grape | Maize |
| Lana | 17 | 14 | 5 | 0.29 | 1 | 0.59 | 4.26 | 1 | 0.039 | 6.55 | 1 | 0.011 | Raisin | Grape | Maize |
| Margarita | 17 | 19 | 0 | 0.11 | 1 | 0.74 | 19.00 | 1 | < 0.001 | 17.00 | 1 | < 0.001 | Grape | Raisin | Maize |
| Matoury | 18 | 14 | 4 | 0.50 | 1 | 0.48 | 5.56 | 1 | 0.018 | 8.91 | 1 | 0.003 | Raisin | Grape | Maize |
| Mekoe | 19 | 14 | 3 | 0.76 | 1 | 0.38 | 7.12 | 1 | 0.008 | 11.64 | 1 | < 0.001 | Raisin | Grape | Maize |
| Pixie | 18 | 15 | 3 | 0.27 | 1 | 0.60 | 8.00 | 1 | 0.005 | 10.71 | 1 | 0.001 | Raisin | Grape | Maize |
| Ruben | 20 | 16 | 0 | 0.44 | 1 | 0.50 | 16.00 | 1 | < 0.001 | 20.00 | 1 | < 0.001 | Raisin | Grape | Maize |
| Rufo | 18 | 12 | 6 | 1.20 | 1 | 0.27 | 2.00 | 1 | 0.16 | 6.00 | 1 | 0.014 | Raisin | Grape | Maize |
| Torres | 20 | 9 | 7 | 4.17 | 1 | 0.04 | 0.25 | 1 | 0.62 | 6.26 | 1 | 0.012 | Raisin | Grape | Maize |
| Ximo | 21 | 14 | 1 | 1.40 | 1 | 0.24 | 11.27 | 1 | < 0.001 | 18.18 | 1 | < 0.001 | Raisin | Grape | Maize |

* If two comparisons (e.g., medium vs. low & high vs. low) were both highly significant (i.e., p< 0.001), food preference was determined based on overall choice counts.

** was given extra session to reach significance.

**Table S4. Digging duration.** Full model outputs for the effects of condition and final food value on digging duration.

|  | Estimate | SE | df | z | *p* | LowerCI | UpperCI | Min | max |
| --- | --- | --- | --- | --- | --- | --- | --- | --- | --- |
| **Intercept** | -1.760 | 0.119 |  |  | ^(2)^ | -2.018 | -1.558 | -1.798 | -1.730 |
| **Condition.Nonmagic ^(1)^** | 0.086 | 0.146 |  |  | ^(2)^ | -0.203 | 0.382 | 0.026 | 0.142 |
| **FinalFoodValue.l ^(1)^** | 0.096 | 0.148 |  |  | ^(2)^ | -0.191 | 0.392 | 0.037 | 0.145 |
| **Condition.Nonmagic: FinalFoodValue.l ^(1)^** | -0.183 | 0.207 | 1 | -0.881 | 0.379 | -0.580 | 0.222 | -0.274 | -0.104 |

^(1)^ Condition and Final food value were dummy coded with ‘magic’ and ‘high’ as the respective reference categories.

^(2)^ not indicated due to limited interpretation

**Table S5. Eating Latency.** Full model output for the effects of condition and final food value on latency to eat the reward.

|  | Estimate | SE | df | t | *p* | LowerCI | UpperCI | min | max |
| --- | --- | --- | --- | --- | --- | --- | --- | --- | --- |
| Intercept | 1.056 | 0.12 |  |  | ^(2)^ | 0.835 | 1.265 | 1.009 | 1.078 |
| Condition.Nonmagic ^(1)^ | 0.015 | 0.161 |  |  | ^(2)^ | -0.276 | 0.314 | -0.01 | 0.05 |
| FinalFoodValue.l ^(1)^ | 0.674 | 0.169 |  |  | ^(2)^ | 0.34 | 0.995 | 0.537 | 0.749 |
| Condition.Nonmagic: FinalFoodValue.l ^(1)^ | -0.418 | 0.243 | 36.574 | -1.72 | 0.094 | -0.894 | 0.084 | -0.578 | -0.227 |

^(1)^ Condition and Final food value were dummy coded with ‘magic’ and ‘high’ as the respective reference categories.

^(2)^ not indicated due to limited interpretation

**Table S6. Eating Latency.** Reduced model output for the effects of condition and final food value on latency to eat the reward.

|  | Estimate | SE | df | t | *p* | LowerCI | UpperCI | min | max |
| --- | --- | --- | --- | --- | --- | --- | --- | --- | --- |
| Intercept | 1.143 | 0.11 |  |  | ^(2)^ | 0.935 | 1.357 | 1.102 | 1.169 |
| Condition.Nonmagic ^(1)^ | -0.15 | 0.126 | 19.22 | -1.265 | 0.221 | -0.402 | 0.072 | -0.236 | -0.087 |
| FinalFoodValue.l ^(1)^ | 0.477 | 0.126 | 37.34 | 3.785 | 0.001 | 0.235 | 0.706 | 0.419 | 0.518 |

^(1)^ Condition and Final food value were dummy coded with ‘magic’ and ‘high’ as the respective reference categories.

^(2)^ not indicated due to limited interpretation

**Table S7. First tower touched.** Full model outputs for the effect of condition (pre-test or post-test) on whether subjects first contacted the violating tower (0 = no, 1 = yes).

|  | Estimate | SE | df | χ^2^ | *p* | LowerCI | UpperCI | min | max |
| --- | --- | --- | --- | --- | --- | --- | --- | --- | --- |
| Intercept | -0.377 | 0.652 |  |  | ^(2)^ | -24.986 | 2.395 | -0.726 | -0.198 |
| PrePost.PostTest ^(1)^ | -0.18 | 0.826 | 1 | 0.047 | 0.828 | -14.092 | 14.092 | -0.638 | 0.237 |

^(1)^ Exploration trial was dummy coded with ‘Pre-test’ being the reference category

^(2)^ not indicated due to very limited interpretation

**Table S8. Proportion violation tower exploration.** Full model outputs for the effect of exploration trial (pre- or post- test trials) and age on proportion of exploration directed toward the violating tower.

|  | Estimate | SE | df | χ^2^ | *p* | LowerCI | UpperCI | min | max |
| --- | --- | --- | --- | --- | --- | --- | --- | --- | --- |
| Intercept | -0.183 | 0.303 |  |  | ^(3)^ | -0.715 | 0.772 | -0.165 | 0.37 |
| PrePost.PostTest ^(1)^ | -0.216 | 0.466 | 1 | 0.214 | 0.644 | -1.11 | 0.708 | -0.571 | -0.014 |
| z.Age ^(2)^ | 0.021 | 0.253 | 1 | 0.007 | 0.934 | -0.469 | 0.52 | -0.242 | 0.324 |

^(1)^ Exploration trial was dummy coded with ‘Pre’ being the reference category

^(2)^ Age was z-transformed to a mean of zero and a standard deviation of one. Mean (sd) of Age was 14.8 (6.66).

^(3)^ not indicated due to limited interpretation

**Table S9. Overall tower exploration.** Full model outputs for the effect of exploration trial (pre- or post- test trials) and age on the duration of exploration toward any tower.

|  | Estimate | SE | z | *p* | LowerCI | UpperCI | min | max |
| --- | --- | --- | --- | --- | --- | --- | --- | --- |
| Intercept | -1.765 | 0.194 |  | ^(3)^ | -2.145 | -1.385 | -1.887 | -1.687 |
| PrePost.PostTest ^(1)^ | 0.117 | 0.26 | -0.451 | 0.652 | -0.392 | 0.626 | -0.018 | 0.246 |
| z.Age ^(2)^ | -0.353 | 0.138 | -2.557 | 0.011 | -0.624 | -0.082 | -0.441 | -0.290 |

^(1)^ Exploration trial was dummy coded with ‘Pre-test’ being the reference category

^(2)^ Age was z-transformed to a mean of zero and a standard deviation of one. Mean (sd) of Age was 14.8 (6.66).

^(3)^ not indicated due to limited interpretation

**Table S10. Emotional behaviors.** Counts of emotional behavior counts among subjects between Violation and NonViolation trials.

|  | Violation | | | NonViolation | | |
| --- | --- | --- | --- | --- | --- | --- |
| Subject | Food eating | Vocalizations | Sandplate roughhousing | Food eating | Vocalizations | Sandplate roughhousing |
| Alba | 2 | 1 | 0 | 2 | 0 | 0 |
| Anita | 1 | 2 | 0 | 1 | 1 | 0 |
| Carlos | 2 | 0 | 1 | 2 | 0 | 0 |
| Cayenne | 2 | 0 | 0 | 2 | 0 | 0 |
| Chico | 2 | 0 | 1 | 2 | 0 | 1 |
| Flojo | 2 | 2 | 0 | 2 | 2 | 0 |
| Fudge | 1 | 3 | 2 | 1 | 3 | 2 |
| Inti | 2 | 0 | 2 | 1 | 0 | 1 |
| Junon | 2 | 0 | 0 | 2 | 0 | 0 |
| Kato | 2 | 0 | 0 | 2 | 0 | 0 |
| Lana | 1 | 1 | 0 | 1 | 1 | 0 |
| Margarita | 2 | 4 | 0 | 1 | 5 | 0 |
| Matoury | 2 | 4 | 1 | 2 | 2 | 0 |
| Mekoe | 2 | 0 | 0 | 2 | 0 | 0 |
| Pixie | 2 | 1 | 0 | 2 | 1 | 0 |
| Ruben | 2 | 1 | 0 | 2 | 0 | 0 |
| Rufo | 1 | 0 | 0 | 1 | 0 | 1 |
| Torres | 2 | 2 | 0 | 2 | 1 | 1 |
| Ximo | 2 | 1 | 1 | 2 | 0 | 1 |

**REFERENCES**

Block, J., & Martin, B. (1955). Predicting the behavior of children under frustration. *The Journal of Abnormal and Social Psychology*, *51*(2), 281–285. <https://doi.org/10.1037/h0047628>

Bräuer, J., & Belger, J. (2018). A ball is not a Kong: Odor representation and search behavior in domestic dogs (*Canis familiaris*) of different education. *Journal of Comparative Psychology*, *132*(2), 189-199. <https://doi.org/10.1037/com0000115>

Pepperberg, I. M., Willner, M. R., & Gravitz, L. B. (1997). Development of Piagetian object permanence in grey parrot (*Psittacus erithacus*). *Journal of Comparative Psychology*, *111*(1), 63-75. <https://doi.org/10.1037/0735-7036.111.1.63>

Schnell, A. K., Loconsole, M., Garcia-Pelegrin, E., Wilkins, C., & Clayton, N. S. (2021). Jays are sensitive to cognitive illusions. *Royal Society Open Science*, *8*(8), 202358. <https://doi.org/10.1098/rsos.202358>

Spelke, E. S., Breinlinger, K., Macomber, J., & Jacobson, K. (1992). Origins of knowledge. *Psychological Review*, *99*(4), 605-632. <https://doi.org/10.1037/0033-295X.99.4.605>

Tinklepaugh, O. L. (1928). An experimental study of representative factors in monkeys. *Journal of Comparative Psychology*, *8*(3), 197-236. <https://doi.org/10.1037/h0075798>

Völter, C. J., Tomašić, A., Nipperdey, L., & Huber, L. (2023). Dogs' expectations about occlusion events: from expectancy violation to exploration. *Proceedings of the Royal Society B*, *290*(2003), 20230696. <https://doi.org/10.1098/rspb.2023.0696>

Wilson, V. A., Bethell, E. J., & Nawroth, C. (2023). The use of gaze to study cognition: limitations, solutions, and applications to animal welfare. *Frontiers in Psychology*, *14*, 1147278. <https://doi.org/10.3389/fpsyg.2023.1147278>
